# Supplementary material for: Fast intensity adaptation enhances the encoding of sound in Drosophila
Source: Nat Commun. 2018 Jan 9;9:134. doi: 10.1038/s41467-017-02453-9 (PMC5760620; doi:10.1038/s41467-017-02453-9)
Supplement: Supplementary file 1 — Supplementary Information [file 41467_2017_2453_MOESM1_ESM.pdf]

Supplementary Figures 1-12

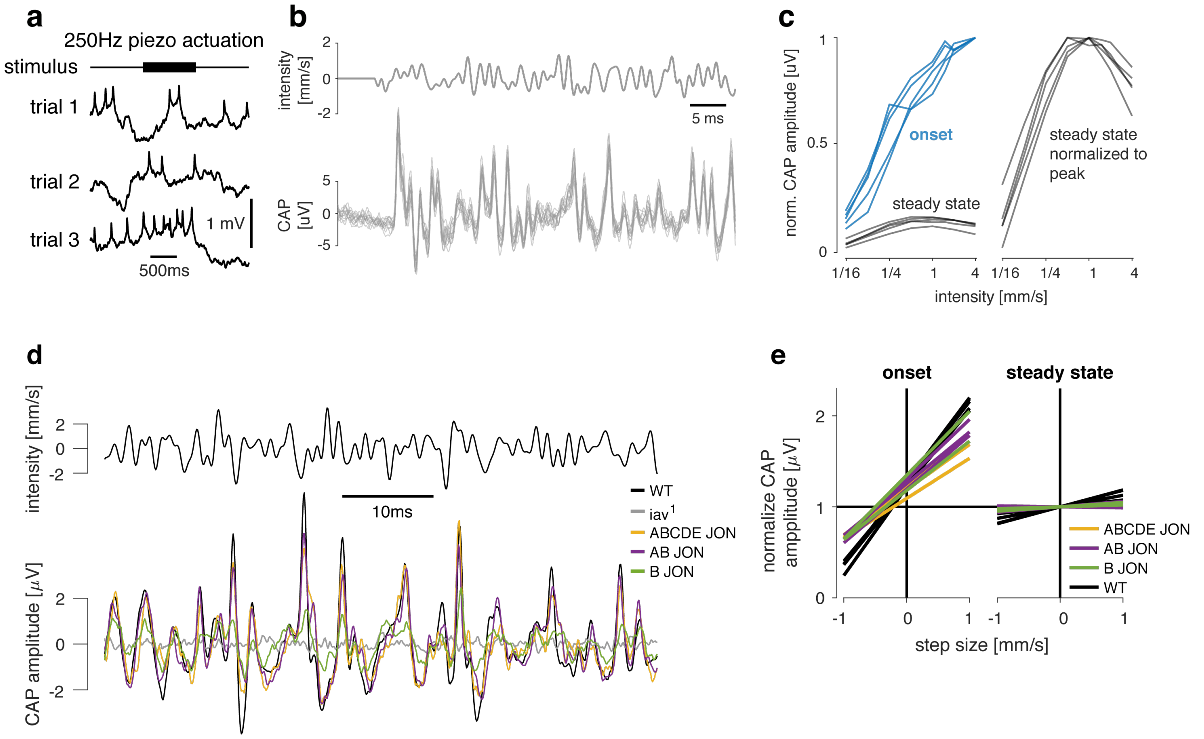

Supplementary Figure 1: *CAP recordings reflect firing of type A and B JON.*

a Patch-clamping interferes with mechanotransduction. Shown are 3 trials of a patch-clamp recording from the cell body of a type AB JON, during which the submerged arista was vibrated via piezoelectric actuation but no consistent, stimulus-evoked change in the membrane voltage was observed (stimulus = 250Hz, upper trace).

b Single-trial responses (bottom) to a noise stimulus (top) are reliable from trial-to-trial.

c Onset (blue, left) and steady-state (black, left) CAP amplitudes for noise responses for individual flies (N=5, averaged over 20 trials each). Curves for each fly were normalized to the maximal onset response. Right panel shows the steady tuning curves individually normalized to their respective maxima.

d CAP responses (bottom) to a white noise stimulus (top) for wild type flies (CS Tully, black), iav^1 mutants (grey) and rescues in all (ABCDE) JONs (yellow), type AB JONs (purple), or type B JONs (green). The iav^1 mutants do not produce CAP responses. CAPs for rescues of all and AB JONs are similar to the wild type. CAPs of type B JONs rescues are weaker and noisier than the other rescues, suggesting that both A and B JON subpopulations contribute to the CAP for white noise stimuli.

e Average onset (left) and steady-state (right) responses for all genotypes in Fig. 1f (N=2-5 flies per genotype, 20 trials each). Reponses are normalized for each fly such that the average steady-state response across step sizes is 1.0. Onset responses depend strongly on intensity and steady-state responses are near intensity invariant, independent of genotype (see legend).

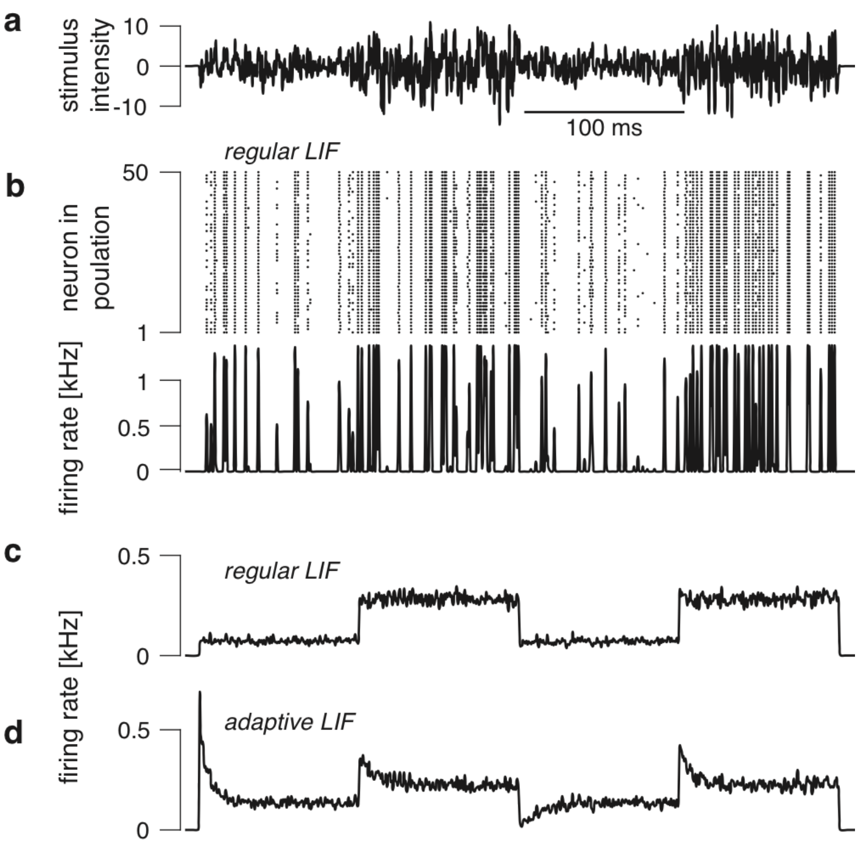

Supplementary Figure 2: *De-synchronization or response heterogeneity in JONs does not explain adaptation dynamics*

We modelled a population of integrate and fire neurons with membrane noise to explore the effects of desynchronization (b-c) and of an adaptation current (d) on firing rate dynamics. For all panels, the neurons in each population had identical parameters and stimulus inputs and only differed in their membrane noise. See Methods for more details on the simulations.

a Noise stimuli used in our study prevent strong de-synchronization in a population of leaky integrate and fire (LIF) neurons. Noise stimulus with zero mean and step-like changes in intensity (switches between 2 and 4mm s^{-1}) as used in the CAP recordings and Ca imaging experiments.

b Responses to the stimulus in a of a population of 500 LIF neurons, each with independent intrinsic noise. Spike raster plot (top, only a subset of 50 neurons shown for clarity) and population firing rate (bottom) do not exhibit de-synchronization at stimulus onset and no transients in synchrony or firing rate for changes in stimulus intensity following stimulus onset. Note that synchrony does change with stimulus intensity through an increase in the signal-to-noise ratio: louder stimuli produce stronger inputs relative to intrinsic noise sources and hence weaker desynchronization. However, this effect is not transient and hence does not explain adaptation.

c Population average firing rate of LIF neurons for 500 independent realizations of the noise stimulus in a. There are no transients in firing rate.

d Same as c but using LIF neurons with an adaptation current. Negative and positive transients arise upon changes in noise intensity just as in our CAP recordings.

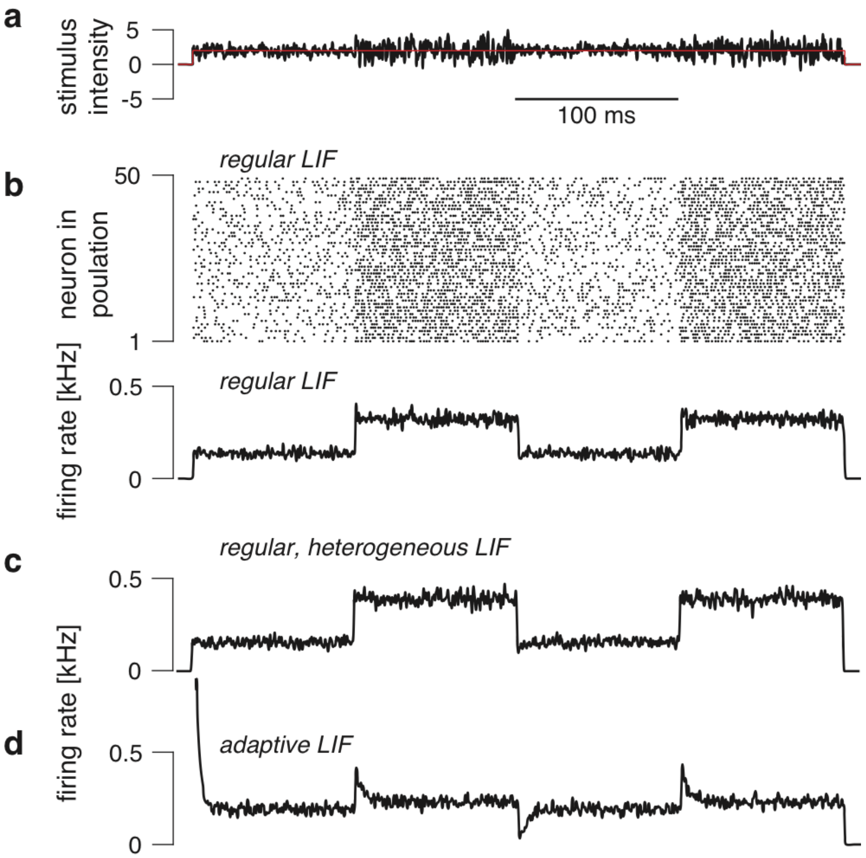

Supplementary Figure 3: *A stimulus regime that promotes de-synchronization or response heterogeneity in JONs does not explain adaptation dynamics*
We modelled a population of integrate and fire neurons with membrane noise to explore the effects of desynchronization (b), response heterogeneity (c), and of an adaptation current (d) on firing rate dynamics. In contrast to the simulations shown in Supplementary Figure 2, we used noise with a constant offset as a stimulus to promote desynchronizations. For all panels except for panel d, the neurons in each population had identical parameters and stimulus inputs and only differed in their membrane noise. For panel d, we explicitly tested the effect of response heterogeneity by choosing the membrane time constant for each neuron in the population at random. See Methods for more details on the simulations.
a Noise stimulus with step-like changes in intensity (switches between 0.5 and 1 mm s^{-1}) with an added 2mm s^{-1} offset that induces a constant depolarization of the model neurons and promotes de-synchronization.
b Responses of a population of LIF neurons for 500 independent realizations of the noise stimulus. Spike raster plot (top, only a subset of 50 neurons shown for clarity) and population firing rate (bottom) lack transients in firing.
c Same as b but for a population of regular LIF neurons with heterogeneous adaptation and integration properties (membrane time constants in each neuron was scaled by a random factor between ½ and 2). The heterogeneity does not produce the strong transient changes in firing rate upon step changes in intensity see on the data.
d Same as b but for LIF neurons with an adaptation current. Adaptation induces transient changes in firing rate – but not in synchrony – upon step changes in intensity. Overall this suggests that neither desynchronization nor population heterogeneity are not sufficient to explain the adaptation dynamics in the CAP and that adaptation is necessary to do so.

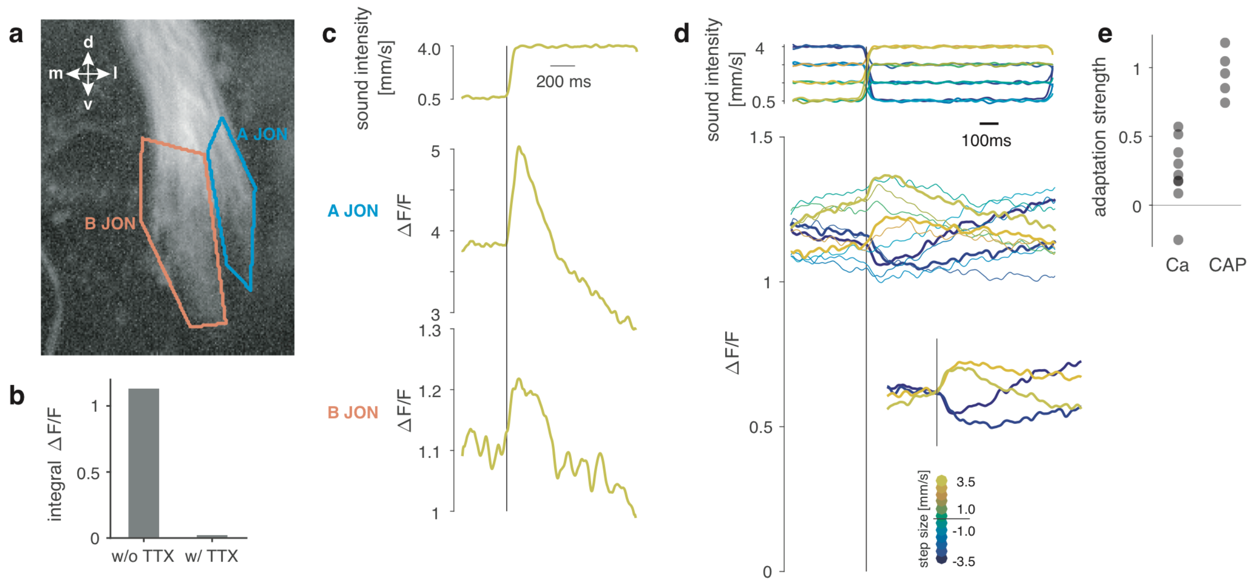

Supplementary Figure 4: *Calcium signals from type A and B JON display adaptation.*

a Type A and B JONs can be distinguished anatomically because they branch separately into the AMMC. Figure shows two-photon baseline fluorescence of JON expressing GCaMP6f under the control of JO15-Gal4.

b Calcium responses (ΔF/F) to 200 Hz sinusoids with and without bath application of TTX (~6 µM). TTX has been shown previously to abolish spiking in JONs [1].

c Representative calcium responses (ΔF/F) to the largest positive step in noise intensity measured in the projections of type A JONs (middle) or type B JONs (bottom) shown in panel a (40 trials per step). The stimulus is similar to that used for the CAP recordings (Fig. 1b) but with an interval between steps of 1000ms to account for the slow dynamics of calcium signals. Both Type A and B JON projections produce transient changes in fluorescence upon step changes in noise intensity, indicating that both subpopulations adapt to sound intensity. These calcium transients are weaker and slower than those seen in the CAP (see Fig. 1), likely due to filtering by the calcium sensor.

d Steps in noise intensity (top) used to probe adaptation in calcium signals. Responses are pooled across type A and B JONs in one fly (bottom). Step size is color-coded (see legend). The calcium signals (bottom) display weak but clear positive and negative transients locked to the time of the intensity step (traces from one fly, averaged over 20 trials per step). This is most evident for the responses to the two most positive and the two most negative steps in noise intensity (thick lines). To highlight the transients, the inset shows responses to the greatest steps in intensity, with baseline subtraction. Changes in fluorescence only weakly depend on absolute noise intensity, consistent with our observation of intensity invariance in the CAP.

e Strength of adaptation was obtained by comparing onset responses to intensity steps to the responses at steady-state (see Methods for details, N=8 flies). Adaptation values of 1.0 indicate complete adaptation. Values for the CAP are reproduced from Fig. 4g.

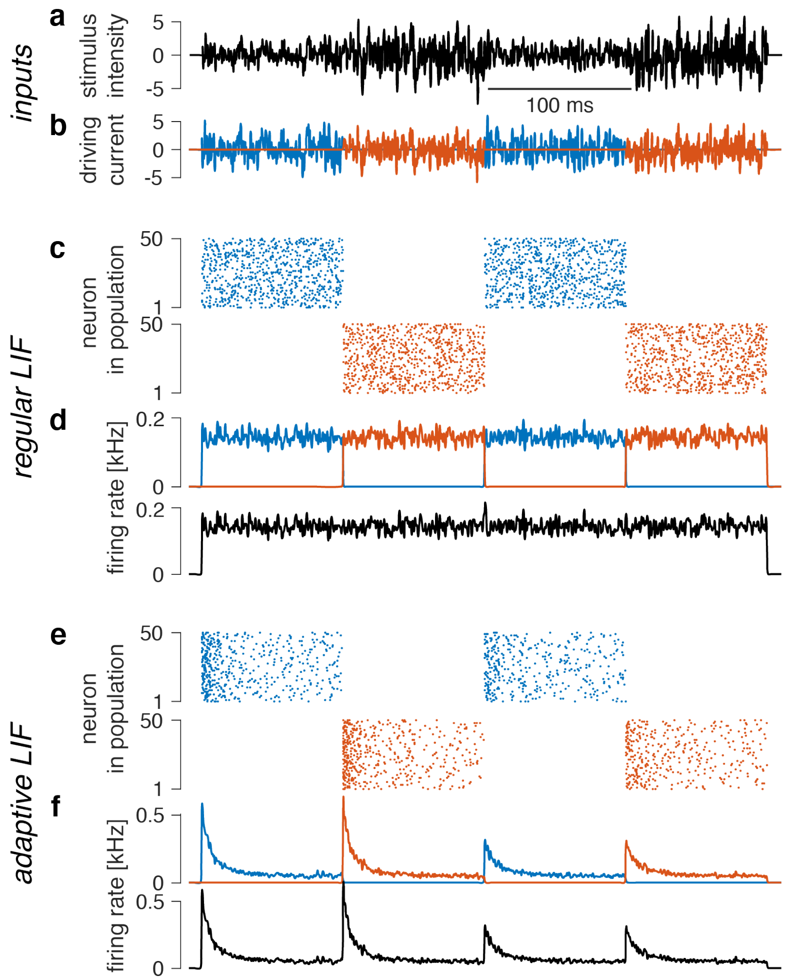

Supplementary Figure 5: *Intensity range fractionation does not explain adaptation dynamics*
Heterogeneity in intensity tuning among individual JO neurons can produce intensity invariance at the population level through range fractionation – individual JONs tiling the relevant intensity range

a Noise stimulus with steps in intensity (1 and 2 mm s^{-1}, top).

b Two populations of neurons that respond exclusively at noise intensities of either 1 (blue) or 2 (orange) mm s^{-1}.

c, d Spike raster plots (c) and population firing rates (d) of the low-intensity (blue) and high-intensity (orange) population of leaky integrate and fire (LIF) neurons. This model only produces transient increases in firing rate upon increases as well as decreases in intensity. By contrast, JONs produce positive transients for intensity increases, and negative transients for intensity decreases (Fig. 1c).

e, f Same as c, d but with adaptive LIF model neurons. Adaptation attenuates the positive transients for the two last steps in noise intensity if the adaptation time constant exceeds the step duration. However, adaptation fails to reproduce the pattern of positive and negative transients in the population firing rate observed in the CAP. Intensity range fractionation – or more generally heterogeneity in intensity tuning among JONs - is thus unlikely to underlie the adaptive dynamics of the CAP. See Methods for details on the parameters of the simulations.

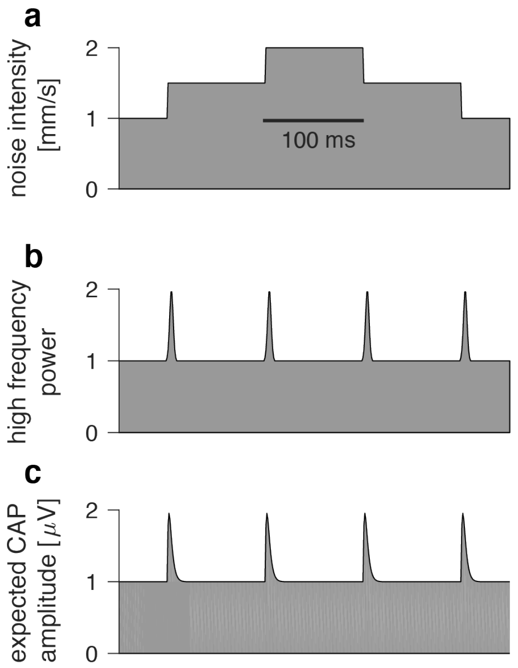

Supplementary Figure 6: *Frequency range fractionation does not explain adaptation dynamics*
While our noise stimulus exhibits a relatively constant frequency spectrum, steps in noise intensity (a) can introduce transient increases in high frequency power (b, schematic) and produce transients in the CAP (c, schematic) by briefly recruiting JON that are tuned for higher frequencies. Known tuning profiles of JON are not narrow enough to produce the strong transient that we see in the CAP [2]. Moreover, the resulting pattern of transients is not consistent with the data (see Fig. 1c), since transients would always positive independent of the direction of the intensity step. We conclude that heterogeneity in frequency tuning cannot explain the CAP dynamics.

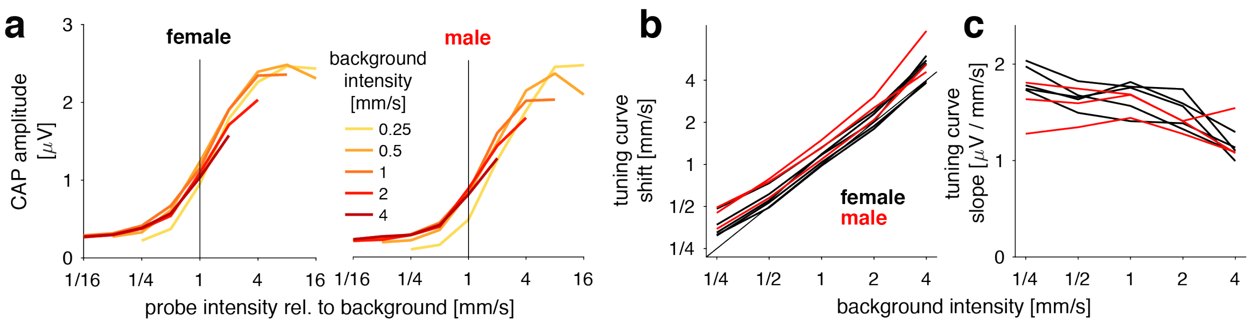

Supplementary Figure 7: *Intensity adaptation is not sex-specific*
a Intensity adaptation is similar in females (left) and males (right). Representative intensity tuning curves for a single fly (averaged over 20 trials) on an intensity scale relative the background intensity (female data reproduced from Fig. 2)
b, c The tuning curve shift (b and slope (c) extracted from sigmoidal fits to the set of tuning curves for different backgrounds for all male and female flies (N=5 females (black), N=3 males (red)). Changes in tuning curve parameters with adaptation are similar for males and females, indicating that adaptation is not sex-specific.

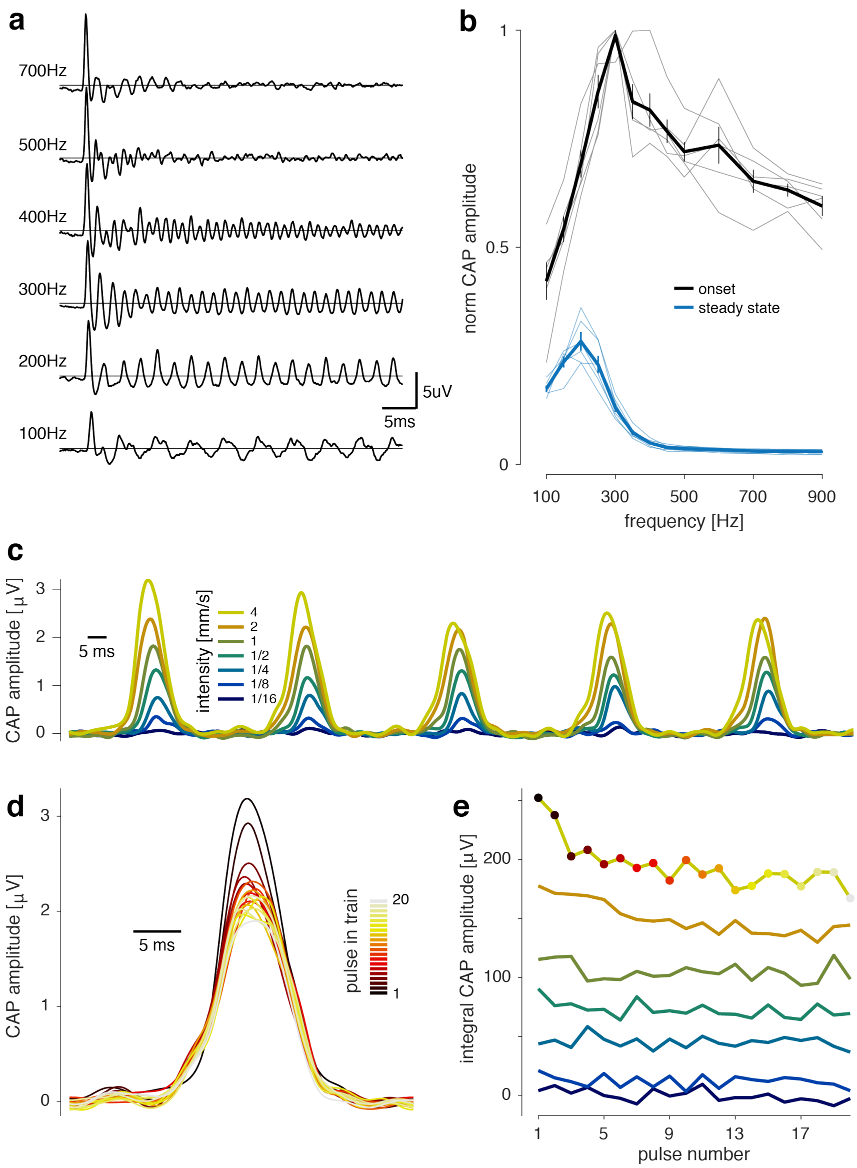

Supplementary Figure 8: *Intensity adaptation for song*

a Pure tones of different frequencies all elicit adaptation in the CAP (data shown for one fly, averaged over 20 trials, sound intensity of 2 mm s^{-1}).

b Onset (black) and steady-state (blue) responses relative to stimulus frequency (thick lines with error bars are mean±std for N=5 flies, 20 trials; thin lines show tuning for individual flies). The onset and steady-state tuning curve for each fly was normalized such that the maximal onset response was 1.0. Onset responses are given by the average CAP amplitude over the first 10 ms of the stimulus. Steady state responses correspond to the CAP amplitude averaged over 100 ms starting 300 ms after stimulus onset.

c CAP amplitude for pulse trains (interval 36 ms) at different intensities (color coded, see legend) for one fly (20 trials).

d Responses to pulses 1 - 20 in a train delivered at 4 mm s^{-1} (same data as in c). Pulse number in train is color coded (see legend).

e CAP amplitude as a function of pulse number for different pulse intensities (color coded, see legend in c). Colored dots correspond to the pulses shown in d.

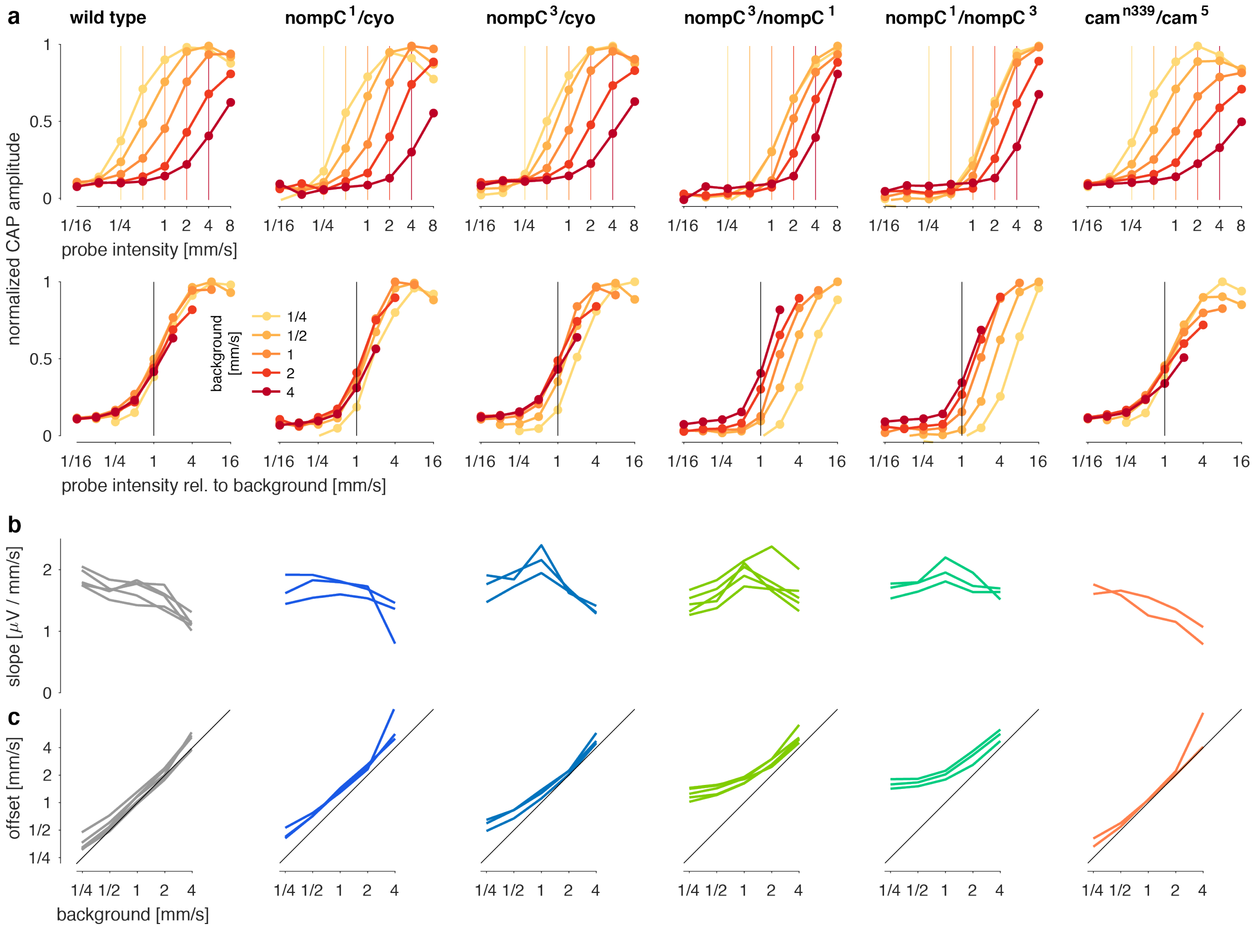

Supplementary Figure 9: *Intensity adaptation persists in nompC and CaM mutants*

a Intensity tuning curves for different adaptation backgrounds (color coded, see legend) on an absolute (top) and a relative (bottom) intensity scale (compare with Figs. 2b-c). The colored vertical lines (top) indicate the respective background intensity, which corresponds to the black vertical line at 1.0 (bottom) on the relative scale. Plots show tuning curves for one fly of each genotype averaged over 20 trials.

b, c Slope (b) and offset (c) extracted from sigmoidal fits to the tuning curves for all flies for each genotype. The diagonal line in c corresponds to a perfect match between tuning curve position and background and implies perfect adaptation. Horizontal curves in c would indicate complete absence of adaptation (no tuning curve shift). Tuning curve slopes (b) cover similar ranges across genotypes. Differences in adaptation (c) between strains can be explained by differences in sound sensitivity: some background intensities evoke no responses, and hence no adaptation. The *nompC* heterozygotes (*nompC^{1}/cyo* and *nompC^{3}/cyo*) exhibit only weakly reduced sensitivity and have largely wild type-like adaptation. In contrast, the *nompC* double mutants (*nompC^{3}/nompC^{1}* and *nompC^{1}/nompC^{3}*) are much less sensitive to sound and their adaptation deviates most strongly from wild type – nonetheless adaptation is still observed: for background intensities that evoke strong responses (2 and 4 mm s^{-1}), adaptation is like that of wild type. For soft backgrounds, tuning curves still shift with background intensity (a) but adaptation is less complete, as indicated by the deviation of the curves from the diagonal line. Wild type (CS Tully) N=5, *nompC^{1}/cyo* N=3, *nompC^{3}/cyo* N=3, *nompC^{3}/nompC^{1}* N=5, *nompC^{1}/nompC^{3}* N=3, *cam^{n339}/cam^{5}* N=2.

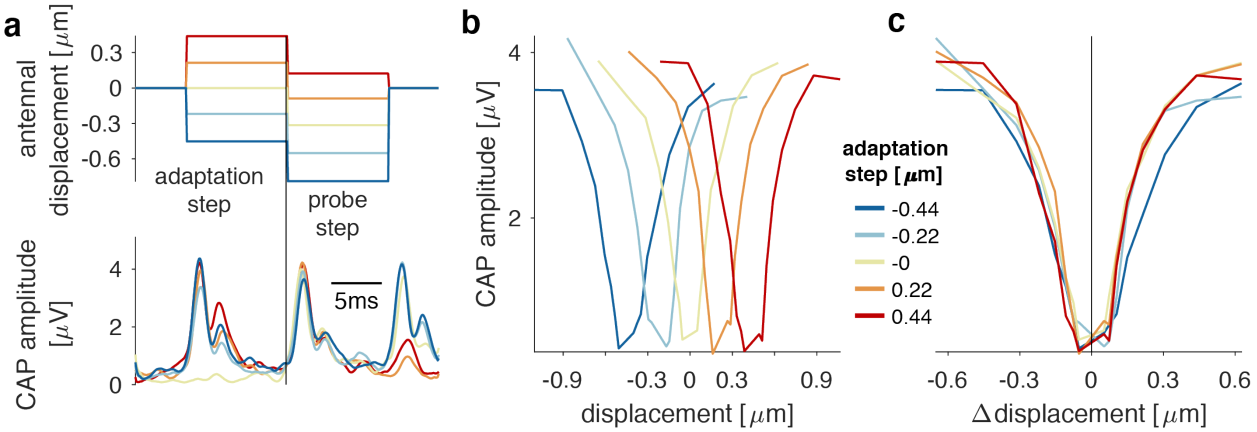

Supplementary Figure 10: *Piezoelectric actuation reproduces offset adaptation.*
a Stimuli for probing offset (mean) adaptation – a piezoelectric actuator was used to step-deflect the antenna to induce offset adaptation (top). Sensitivity to subsequent steps was estimated by measuring the CAP amplitude (bottom; color coded – see legend insert in b).
b Step tuning curves for different levels of mean adaptation (color coded, see legend). Tuning curve values correspond to the CAP amplitude of the first response peak after the probe step (a)
c Same curves as in b, with the adaptation background subtracted from the displacement values. All curves overlap, suggesting that JONs encode displacement relative to an additive offset (mean).
All plots show responses for one representative fly, averaged over 40 trials.

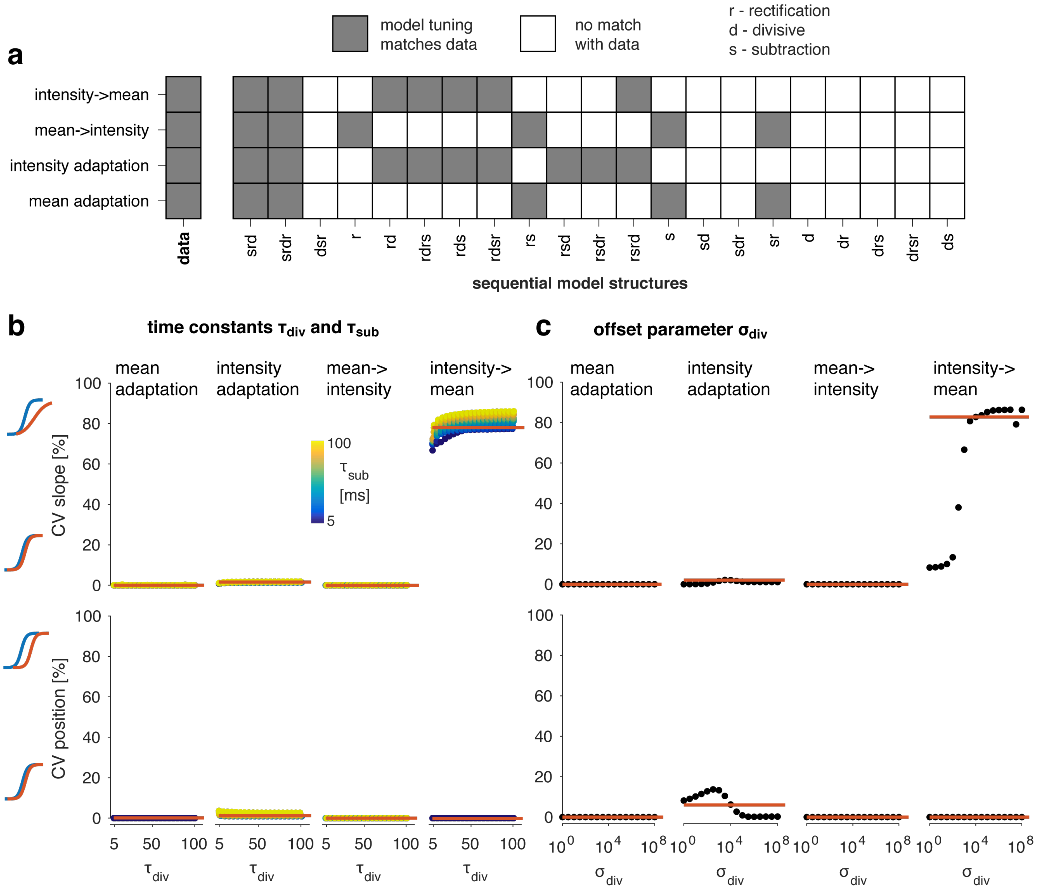

Supplementary Figure 11: *A phenomenological model of mean and variance adaptation*
a Match between model and data for all models tested. Rows correspond to different adaptation paradigms: The impact of intensity adaptation on step responses (first row), the impact of mean adaptation on intensity tuning (second row), the impact of intensity adaptation or of mean adaptation (3rd and 4th rows). A gray box indicates a match, a white box a mismatch, between model and data. Only two computational motifs (the first two columns) can reproduce our data – both correspond to a sequence of subtractive adaptation (S), rectification (R) and divisive adaptation (D). SRD and SRDR are equivalent – the final rectification step in SRDR is redundant and does not affect model output.
b, c Model behavior is robust to changes in the time constants for the subtractive and divisive adaptation stage τ_{sub} and τ_{div} (**b**, $\sigma_{div}=10^{-4}$) or the adaptation strength σ_{div} of the divisive adaptation stage (**c**, τ_{sub}=30 ms, τ_{div}=50 ms). For **b**, τ_{div} are plotted as x-values, while τ_{sub} is color-coded (see legend). Since model behavior is relatively independent of τ_{sub}, points for different values of τ_{sub} overlap. Columns in **b** and **c** correspond to different adaptation paradigms: 1) mean adaptation, 2) intensity adaptation, 3) the effect of mean adaptation on intensity tuning, and 4) the effect of intensity adaptation on step responses. Model behavior was quantified using the coefficient of variation (CV) across different adaptation states (e.g. adaptation tone intensity or adaptation step size) for tuning curve slope (top) and position (bottom) extracted from sigmoidal fits (see Methods for details) – pictograms to the left of **b** illustrate tuning curves corresponding to the CVs for a given parameter. The red line corresponds to the CV values obtained for the reference model shown in Fig. 6 that matches our JO data. Most parameter combinations tested produced adaptation like that of the reference model. For small values of the adaptation strength σ_{div} adaptation is too weak to affect tuning curve slope. The model behavior is thus a general property of the computational motif, not the outcome of the specific parameters chosen.

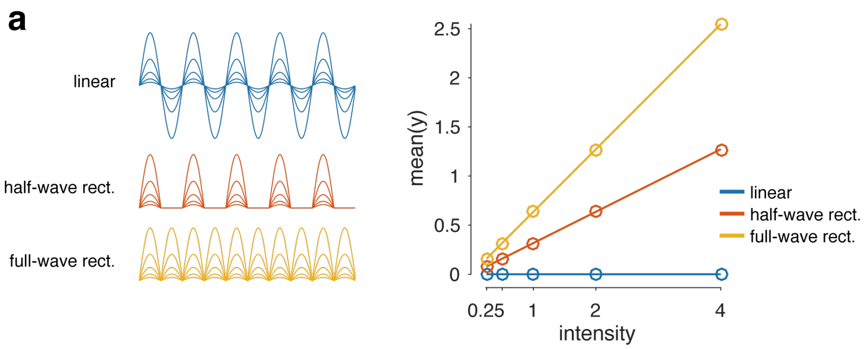

Supplementary Figure 12: *Half-wave and full-wave rectification encode stimulus intensity*
a A sinusoidal signal of different intensities (left, blue), and the outcome of half-wave rectification (red) or full-wave rectification (orange). The graph shows the mean of the three signals. The mean of the original sinusoidal is zero due to the symmetry of the signal. Full-wave or half-wave rectification lead to the signal mean encoding input magnitude (sound intensity), which is a pre-requisite for variance adaptation in the model.

Supplementary References

1. Lehnert, B. P., Baker, A. E., Gaudry, Q., Chiang, A.-S. & Wilson, R. I. Distinct Roles of TRP Channels in Auditory Transduction and Amplification in Drosophila. *Neuron* **77,** 115–128 (2013).
2. Ishikawa, Y., Okamoto, N., Nakamura, M., Kim, H. & Kamikouchi, A. Anatomic and Physiologic Heterogeneity of Subgroup-A Auditory Sensory Neurons in Fruit Flies. *Front. Neural Circuits* **11,** 46 (2017).
