# Supplementary material for: Fast intensity adaptation enhances the encoding of sound in Drosophila
Source: Nat Commun. 2018 Jan 9;9:134. doi: 10.1038/s41467-017-02453-9 (PMC5760620; doi:10.1038/s41467-017-02453-9)
Supplement: Supplementary file 2 — Peer Review File [file 41467_2017_2453_MOESM2_ESM.pdf]

Editorial Note: *this manuscript has been previously reviewed at another journal that is not operating a transparent peer review scheme. This document only contains reviewer comments and rebuttal letters for versions considered at Nature Communications.*
